# Supplementary material for: Modeling glioblastoma heterogeneity as a dynamic network of cell states
Source: Mol Syst Biol. 2021 Sep 16;17(9):e10105. doi: 10.15252/msb.202010105 (PMC8444284; doi:10.15252/msb.202010105)
Supplement: Supplementary file 5 — Source Data for Figure 3 [file MSB-17-e10105-s001.zip › Figure3A_sourcedata/GSEA_3065/hallmarks_state5.GseaPreranked.1623416302988/HALLMARK_ADIPOGENESIS.html]

Details for gene set HALLMARK\_ADIPOGENESIS[GSEA]

|  || Dataset | state5 |
| Phenotype | NoPhenotypeAvailable |
| Upregulated in class | na\_neg |
| GeneSet | HALLMARK\_ADIPOGENESIS |
| Enrichment Score (ES) | -0.40578404 |
| Normalized Enrichment Score (NES) | -1.470275 |
| Nominal p-value | 0.0061500617 |
| FDR q-value | 0.058323186 |
| FWER p-Value | 0.488 |
Table: GSEA Results Summary

  

Fig 1: Enrichment plot: HALLMARK\_ADIPOGENESIS      
 Profile of the Running ES Score & Positions of GeneSet Members on the Rank Ordered List

  

| PROBE | GENE SYMBOL | GENE\_TITLE | RANK IN GENE LIST | RANK METRIC SCORE | RUNNING ES | CORE ENRICHMENT || 1 | APLP2 |  |  | 31 | 0.733 | 0.0192 | No |
| 2 | COL4A1 |  |  | 54 | 0.634 | 0.0364 | No |
| 3 | ATP1B3 |  |  | 113 | 0.510 | 0.0457 | No |
| 4 | RTN3 |  |  | 123 | 0.505 | 0.0603 | No |
| 5 | ITGA7 |  |  | 207 | 0.436 | 0.0644 | No |
| 6 | POR |  |  | 215 | 0.430 | 0.0769 | No |
| 7 | CD151 |  |  | 243 | 0.412 | 0.0867 | No |
| 8 | DHCR7 |  |  | 387 | 0.347 | 0.0813 | No |
| 9 | REEP5 |  |  | 489 | 0.315 | 0.0796 | No |
| 10 | HIBCH |  |  | 585 | 0.289 | 0.0778 | No |
| 11 | LPL |  |  | 646 | 0.276 | 0.0796 | No |
| 12 | ABCB8 |  |  | 665 | 0.272 | 0.0860 | No |
| 13 | DHRS7B |  |  | 700 | 0.262 | 0.0902 | No |
| 14 | ADCY6 |  |  | 782 | 0.245 | 0.0887 | No |
| 15 | IDH1 |  |  | 793 | 0.242 | 0.0950 | No |
| 16 | ESYT1 |  |  | 896 | 0.227 | 0.0905 | No |
| 17 | PHLDB1 |  |  | 906 | 0.224 | 0.0964 | No |
| 18 | LAMA4 |  |  | 931 | 0.220 | 0.1006 | No |
| 19 | IFNGR1 |  |  | 983 | 0.213 | 0.1014 | No |
| 20 | ELMOD3 |  |  | 1052 | 0.204 | 0.1000 | No |
| 21 | PPP1R15B |  |  | 1156 | 0.191 | 0.0942 | No |
| 22 | SLC19A1 |  |  | 1198 | 0.185 | 0.0953 | No |
| 23 | DHRS7 |  |  | 1239 | 0.181 | 0.0964 | No |
| 24 | DGAT1 |  |  | 1398 | 0.162 | 0.0835 | No |
| 25 | SLC27A1 |  |  | 1438 | 0.158 | 0.0840 | No |
| 26 | LIFR |  |  | 1520 | 0.150 | 0.0795 | No |
| 27 | SPARCL1 |  |  | 1663 | 0.136 | 0.0677 | No |
| 28 | BCL6 |  |  | 1664 | 0.136 | 0.0719 | No |
| 29 | ATL2 |  |  | 1683 | 0.135 | 0.0740 | No |
| 30 | MIGA2 |  |  | 1711 | 0.133 | 0.0751 | No |
| 31 | ELOVL6 |  |  | 1914 | 0.117 | 0.0558 | No |
| 32 | PTCD3 |  |  | 1969 | 0.114 | 0.0533 | No |
| 33 | DNAJB9 |  |  | 1987 | 0.112 | 0.0548 | No |
| 34 | ABCA1 |  |  | 2142 | 0.103 | 0.0406 | No |
| 35 | LPCAT3 |  |  | 2343 | 0.089 | 0.0207 | No |
| 36 | SLC5A6 |  |  | 2348 | 0.089 | 0.0230 | No |
| 37 | DRAM2 |  |  | 2469 | 0.081 | 0.0119 | No |
| 38 | ANGPT1 |  |  | 2651 | 0.071 | -0.0064 | No |
| 39 | ACOX1 |  |  | 2700 | 0.068 | -0.0097 | No |
| 40 | JAGN1 |  |  | 2752 | 0.065 | -0.0135 | No |
| 41 | SCARB1 |  |  | 2808 | 0.062 | -0.0178 | No |
| 42 | BAZ2A |  |  | 2920 | 0.057 | -0.0286 | No |
| 43 | DBT |  |  | 3182 | 0.042 | -0.0568 | No |
| 44 | GPAT4 |  |  | 3216 | 0.040 | -0.0593 | No |
| 45 | CCNG2 |  |  | 3262 | 0.038 | -0.0632 | No |
| 46 | NKIRAS1 |  |  | 3338 | 0.034 | -0.0706 | No |
| 47 | PPM1B |  |  | 3347 | 0.034 | -0.0705 | No |
| 48 | MAP4K3 |  |  | 3376 | 0.032 | -0.0727 | No |
| 49 | RMDN3 |  |  | 3398 | 0.032 | -0.0740 | No |
| 50 | ADIPOR2 |  |  | 3460 | 0.029 | -0.0801 | No |
| 51 | GPD2 |  |  | 3594 | 0.024 | -0.0944 | No |
| 52 | STOM |  |  | 3625 | 0.023 | -0.0971 | No |
| 53 | AGPAT3 |  |  | 3684 | 0.021 | -0.1030 | No |
| 54 | PEMT |  |  | 3834 | 0.014 | -0.1194 | No |
| 55 | GPAM |  |  | 3968 | 0.008 | -0.1342 | No |
| 56 | IMMT |  |  | 3978 | 0.008 | -0.1350 | No |
| 57 | MCCC1 |  |  | 4042 | 0.006 | -0.1420 | No |
| 58 | SORBS1 |  |  | 4146 | 0.001 | -0.1536 | No |
| 59 | GBE1 |  |  | 4323 | -0.007 | -0.1733 | No |
| 60 | ARAF |  |  | 4338 | -0.007 | -0.1747 | No |
| 61 | PIM3 |  |  | 4517 | -0.015 | -0.1944 | No |
| 62 | SSPN |  |  | 4582 | -0.017 | -0.2011 | No |
| 63 | CPT2 |  |  | 4804 | -0.026 | -0.2253 | No |
| 64 | ACO2 |  |  | 4811 | -0.026 | -0.2252 | No |
| 65 | TST |  |  | 4856 | -0.028 | -0.2293 | No |
| 66 | ALDH2 |  |  | 4969 | -0.032 | -0.2410 | No |
| 67 | TOB1 |  |  | 5047 | -0.035 | -0.2486 | No |
| 68 | SAMM50 |  |  | 5258 | -0.043 | -0.2711 | No |
| 69 | COX6A1 |  |  | 5281 | -0.043 | -0.2723 | No |
| 70 | ESRRA |  |  | 5336 | -0.046 | -0.2769 | No |
| 71 | CAT |  |  | 5358 | -0.047 | -0.2779 | No |
| 72 | DNAJC15 |  |  | 5399 | -0.048 | -0.2809 | No |
| 73 | DLAT |  |  | 5529 | -0.054 | -0.2938 | No |
| 74 | COQ3 |  |  | 5559 | -0.055 | -0.2954 | No |
| 75 | ITSN1 |  |  | 5686 | -0.062 | -0.3077 | No |
| 76 | TANK |  |  | 5756 | -0.064 | -0.3136 | No |
| 77 | FAH |  |  | 5845 | -0.067 | -0.3215 | No |
| 78 | SLC25A10 |  |  | 5851 | -0.067 | -0.3199 | No |
| 79 | IDH3G |  |  | 5901 | -0.070 | -0.3233 | No |
| 80 | ALDOA |  |  | 5909 | -0.070 | -0.3219 | No |
| 81 | AIFM1 |  |  | 6081 | -0.078 | -0.3389 | No |
| 82 | PLIN2 |  |  | 6129 | -0.081 | -0.3417 | No |
| 83 | SDHC |  |  | 6193 | -0.085 | -0.3462 | No |
| 84 | CS |  |  | 6219 | -0.086 | -0.3463 | No |
| 85 | PDCD4 |  |  | 6228 | -0.086 | -0.3446 | No |
| 86 | PHYH |  |  | 6255 | -0.088 | -0.3448 | No |
| 87 | PREB |  |  | 6305 | -0.090 | -0.3475 | No |
| 88 | COQ5 |  |  | 6330 | -0.092 | -0.3474 | No |
| 89 | RIOK3 |  |  | 6373 | -0.094 | -0.3493 | No |
| 90 | NMT1 |  |  | 6545 | -0.105 | -0.3654 | No |
| 91 | IDH3A |  |  | 6585 | -0.107 | -0.3665 | No |
| 92 | BCL2L13 |  |  | 6736 | -0.116 | -0.3799 | No |
| 93 | CRAT |  |  | 6922 | -0.131 | -0.3968 | No |
| 94 | UCK1 |  |  | 6925 | -0.131 | -0.3929 | No |
| 95 | ARL4A |  |  | 6930 | -0.131 | -0.3893 | No |
| 96 | UBC |  |  | 6969 | -0.135 | -0.3894 | No |
| 97 | MGLL |  |  | 7100 | -0.145 | -0.3996 | No |
| 98 | HADH |  |  | 7129 | -0.148 | -0.3982 | No |
| 99 | PFKL |  |  | 7197 | -0.155 | -0.4010 | Yes |
| 100 | MYLK |  |  | 7203 | -0.155 | -0.3967 | Yes |
| 101 | COQ9 |  |  | 7205 | -0.156 | -0.3920 | Yes |
| 102 | ACLY |  |  | 7208 | -0.156 | -0.3874 | Yes |
| 103 | GRPEL1 |  |  | 7262 | -0.163 | -0.3883 | Yes |
| 104 | PGM1 |  |  | 7268 | -0.164 | -0.3838 | Yes |
| 105 | DLD |  |  | 7363 | -0.176 | -0.3890 | Yes |
| 106 | UBQLN1 |  |  | 7383 | -0.179 | -0.3856 | Yes |
| 107 | NDUFS3 |  |  | 7530 | -0.195 | -0.3961 | Yes |
| 108 | CMBL |  |  | 7534 | -0.195 | -0.3904 | Yes |
| 109 | ACADM |  |  | 7551 | -0.198 | -0.3861 | Yes |
| 110 | SLC25A1 |  |  | 7589 | -0.203 | -0.3840 | Yes |
| 111 | GADD45A |  |  | 7633 | -0.208 | -0.3824 | Yes |
| 112 | AK2 |  |  | 7671 | -0.213 | -0.3800 | Yes |
| 113 | CMPK1 |  |  | 7698 | -0.218 | -0.3762 | Yes |
| 114 | UCP2 |  |  | 7726 | -0.222 | -0.3723 | Yes |
| 115 | MGST3 |  |  | 7746 | -0.225 | -0.3675 | Yes |
| 116 | RNF11 |  |  | 7796 | -0.234 | -0.3658 | Yes |
| 117 | SDHB |  |  | 7809 | -0.236 | -0.3598 | Yes |
| 118 | PRDX3 |  |  | 8005 | -0.277 | -0.3733 | Yes |
| 119 | ACAA2 |  |  | 8017 | -0.279 | -0.3659 | Yes |
| 120 | DECR1 |  |  | 8034 | -0.282 | -0.3590 | Yes |
| 121 | MTCH2 |  |  | 8083 | -0.294 | -0.3553 | Yes |
| 122 | CDKN2C |  |  | 8091 | -0.295 | -0.3470 | Yes |
| 123 | QDPR |  |  | 8112 | -0.299 | -0.3400 | Yes |
| 124 | TKT |  |  | 8122 | -0.301 | -0.3316 | Yes |
| 125 | NDUFA5 |  |  | 8141 | -0.307 | -0.3242 | Yes |
| 126 | G3BP2 |  |  | 8144 | -0.308 | -0.3148 | Yes |
| 127 | UQCRC1 |  |  | 8149 | -0.309 | -0.3057 | Yes |
| 128 | CYC1 |  |  | 8152 | -0.309 | -0.2963 | Yes |
| 129 | MRPL15 |  |  | 8184 | -0.321 | -0.2899 | Yes |
| 130 | SUCLG1 |  |  | 8202 | -0.327 | -0.2817 | Yes |
| 131 | RAB34 |  |  | 8218 | -0.330 | -0.2731 | Yes |
| 132 | ECH1 |  |  | 8238 | -0.335 | -0.2649 | Yes |
| 133 | YWHAG |  |  | 8311 | -0.357 | -0.2620 | Yes |
| 134 | CHCHD10 |  |  | 8332 | -0.363 | -0.2530 | Yes |
| 135 | NDUFAB1 |  |  | 8333 | -0.363 | -0.2417 | Yes |
| 136 | VEGFB |  |  | 8352 | -0.370 | -0.2323 | Yes |
| 137 | TALDO1 |  |  | 8393 | -0.386 | -0.2249 | Yes |
| 138 | GHITM |  |  | 8408 | -0.390 | -0.2144 | Yes |
| 139 | ECHS1 |  |  | 8411 | -0.391 | -0.2025 | Yes |
| 140 | DDT |  |  | 8511 | -0.442 | -0.2000 | Yes |
| 141 | SCP2 |  |  | 8513 | -0.442 | -0.1864 | Yes |
| 142 | NDUFB7 |  |  | 8556 | -0.463 | -0.1768 | Yes |
| 143 | ETFB |  |  | 8610 | -0.499 | -0.1673 | Yes |
| 144 | COX7B |  |  | 8614 | -0.502 | -0.1521 | Yes |
| 145 | CAVIN1 |  |  | 8657 | -0.531 | -0.1404 | Yes |
| 146 | MDH2 |  |  | 8733 | -0.593 | -0.1305 | Yes |
| 147 | ATP5PO |  |  | 8747 | -0.609 | -0.1131 | Yes |
| 148 | UQCR11 |  |  | 8804 | -0.655 | -0.0991 | Yes |
| 149 | UQCR10 |  |  | 8843 | -0.703 | -0.0816 | Yes |
| 150 | SOD1 |  |  | 8848 | -0.709 | -0.0601 | Yes |
| 151 | COX8A |  |  | 8862 | -0.724 | -0.0391 | Yes |
| 152 | UQCRQ |  |  | 8887 | -0.754 | -0.0185 | Yes |
| 153 | GPX4 |  |  | 8966 | -0.946 | 0.0020 | Yes |
Table: GSEA details [plain text format]

  

Fig 2: HALLMARK\_ADIPOGENESIS: Random ES distribution      
 Gene set null distribution of ES for **HALLMARK\_ADIPOGENESIS**
